# Age, vascular disease, and Alzheimer’s disease pathologies in amyloid negative elderly adults

**DOI:** 10.1186/s13195-021-00913-5

**Published:** 2021-10-15

**Authors:** Tengfei Guo, Susan M. Landau, William J. Jagust, for the Alzheimer’s Disease Neuroimaging Initiative

**Affiliations:** 1 Institute of Biomedical Engineering, Shenzhen Bay Laboratory, No.5 Kelian Road, Shenzhen, 518132 China; 2 Helen Wills Neuroscience Institute, University of California, Berkeley, CA 94720 USA; 3 Molecular Biophysics and Integrated Bioimaging, Lawrence Berkeley National Laboratory, Berkeley, CA 94720 USA

**Keywords:** Alzheimer’s disease, Amyloid negative, CSF p-Tau, Hippocampal atrophy, Hypometabolism, Vascular disease

## Abstract

**Background:**

We recently reported that CSF phosphorylated tau (p-Tau_181_) relative to Aβ_40_ (CSF p-Tau/Aβ_40_ ratio) was less noisy and increased associations with Alzheimer’s disease (AD) biomarkers compared to CSF p-Tau_181_ alone. While elevations of CSF p-Tau/Aβ_40_ can occur in amyloid-β (Aβ) negative (Aβ-) individuals, the factors associated with these elevations and their role in neurodegeneration and cognitive decline are unknown. We aim to explore factors associated with elevated tau in CSF, and how these elevated tau are related to neurodegeneration and cognitive decline in the absence of Aβ positivity.

**Methods:**

We examined relationships between CSF p-Tau/Aβ_40_, and CSF Aβ_42_/Aβ_40_, Aβ PET, and white matter hyperintensities (WMH) as well as vascular risk factors in 149 cognitively unimpaired and 52 impaired individuals who were presumably not on the Alzheimer’s disease (AD) pathway due to negative Aβ status on both CSF and PET. Subgroups had ^18^F-fluorodeoxyglucose (FDG) PET and adjusted hippocampal volume (aHCV), and longitudinal measures of CSF, aHCV, FDG PET, and cognition data, so we examined CSF p-Tau/Aβ_40_ associations with these measures as well.

**Results:**

Elevated CSF p-Tau/Aβ_40_ was associated with older age, male sex, greater WMH, and hypertension as well as a pattern of hippocampal atrophy and temporoparietal hypometabolism characteristic of AD. Lower CSF Aβ_42_/Aβ_40_, higher WMH, and hypertension but not age, sex, Aβ PET, APOE-ε4 status, body mass index, smoking, and hyperlipidemia at baseline predicted CSF p-Tau/Aβ_40_ increases over approximately 5 years of follow-up. The relationship between CSF p-Tau/Aβ_40_ and subsequent cognitive decline was partially or fully explained by neurodegenerative measurements.

**Conclusions:**

These data provide surprising clues as to the etiology and significance of tau pathology in the absence of Aβ. It seems likely that, in addition to age, both cerebrovascular disease and subthreshold levels of Aβ are related to this tau accumulation. Crucially, this phenotype of CSF tau elevation in amyloid-negative individuals share features with AD such as a pattern of metabolic decline and regional brain atrophy.

**Supplementary Information:**

The online version contains supplementary material available at 10.1186/s13195-021-00913-5.

## Background

Amyloid-β (Aβ) plaques and tau neurofibrillary tangles are the core features of Alzheimer’s disease (AD) [1]. According to the NIA-AA research framework [2], individuals with evidence of Aβ pathology are on the Alzheimer’s disease continuum, while Aβ-negative (Aβ−) individuals who have abnormal tau and/or neurodegeneration have been regarded as harboring non-Alzheimer’s pathologic change (also known as suspected non-Alzheimer’s pathophysiology or SNAP). Neuropathologically, Aβ- individuals with mild/moderate tau pathology have been characterized as having Primary-Age Related Pathology (PART) [3]. Previous studies [4–8] suggest that medial temporal lobe (MTL) tau aggregation may occur in the absence of abnormal Aβ pathology while Aβ pathology may be involved in driving the spread of tau out of MTL regions, producing neocortical neurodegeneration, and global cognitive decline. On the AD continuum, it is likely that elevated Aβ pathology alone may be insufficient to result in global brain atrophy, cortical hypometabolism, and generalized cognitive decline, requiring this spread of tau to induce these global and neocortical abnormalities [9–13]. This series of events raises the question of whether Aβ is necessary for these downstream events. Recent work has suggested that while elevated MTL tau may be related to local atrophy and cognitive decline regardless of Aβ status [14–16], evidence of cortical hypometabolism appears to require elevated Aβ [17, 18].

While PET provides spatial information on where tau deposits, CSF measurement of phosphorylated tau (p-Tau) provides complementary, although not interchangeable [19–21], information. Our laboratory [21] and other groups [19, 20] very recently observed evidence that CSF p-Tau may be superior to tau PET for detection of early tau increase. Recent data suggests that CSF p-Tau may start to increase even in those who are Aβ- by PET [20, 22]. However, the significance of elevated CSF p-Tau in the absence of Aβ, and how it relates to neurodegeneration, cognitive decline, and other factors, remains unclear. Previous studies in Aβ- individuals have reported inconsistent relationships between CSF p-Tau, neurodegeneration [23–25], and cognition [26–28].

Recently, we reported that using a CSF p-Tau/Aβ_40_ ratio reduced measurement error likely related to individual differences in CSF production rather than pathology and improved associations with AD biomarkers compared to using CSF p-Tau alone [21]. Specifically, we found that normalizing CSF p-Tau by Aβ_40_ eliminated a linear positive relationship otherwise observed between CSF p-Tau and Aβ_42_ among individuals with high (normal) Aβ_42_. This positive association (increasingly abnormal tau as Aβ_42_ becomes elevated or less abnormal within the high Aβ_42_ range) appears to reflect variability in CSF production and not a physiologically meaningful relationship. CSF p-Tau/Aβ_40_ therefore appears to increase sensitivity to detect tau-related neurodegeneration and cognitive decline compared to CSF p-Tau alone, particularly within the low, relatively restricted range of tau measurements observed in Aβ- individuals.

Relationships between tau and a number of other variables have been reported, including age [29–33], sex [33, 34], apolipoprotein E (APOE) genotype [31, 35, 36], and vascular risk factors including white matter hyperintensities (WMH) [25, 37, 38] and blood pressure [39–41]. The question of how these factors relate to tau among Aβ- individuals who are not (yet) on the AD pathway is not fully understood. Exploration of relationships between CSF p-Tau and non-AD-specific risk factors such as WMH, neurodegeneration, and cognitive decline in individuals who are not yet on the AD pathway provide insight into the heterogeneity of AD and other neurodegenerative diseases. In this study, we examined Alzheimer’s disease neuroimaging initiative (ADNI) participants who were unambiguously Aβ- based on both CSF Aβ_42_/Aβ_40_ and Aβ PET biomarkers in order to investigate how age, sex, APOE-ε4, and vascular risk factors associate with the earliest detectable CSF tau cross-sectionally and longitudinally, and whether elevation of CSF p-Tau can predict longitudinal hippocampal atrophy, hypometabolism, and cognitive decline.

## Methods

### Participants

Data used in this study were obtained from the ADNI database (ida.loni.usc.edu). The ADNI study was approved by institutional review boards of all participating centers, and written informed consent was obtained from all participants or their authorized representatives. We identified 150 cognitively unimpaired (CU) participants, and 53 cognitively impaired (CI) participants (51 mild cognitive impairment (MCI) and 2 AD patients) who were Aβ- at baseline on both CSF Aβ_42_/Aβ_40_ and Aβ PET using CSF and PET thresholds described below, and had concurrent (acquisition intervals within 1 year) Aβ PET (^18^F-florbetapir (FBP) or ^18^F-florbetaben (FBB)), CSF Aβ_40_, Aβ_42_, and p-Tau_181_, WMH measurements, vascular risk factor data, and the ADNI cognitive test battery. Notably, one CU and one MCI individual whose WMHs were 3 and 4 standard deviations (SD) below the mean of the sample were excluded from the analysis. In addition, 81 individuals had concurrent ^18^F-fluorodeoxyglucose (FDG) PET and hippocampal volume.

### Vascular risk factors

﻿Based on the vascular risk factor data that is available in ADNI [42], we selected body mass index (BMI), smoking history, diabetes, hyperlipidemia (HLD), and hypertension (HTN). BMI was calculated according to the formula: BMI = (body weight in kg)/(body height in meters^2^). To define smoking, diabetes, HLD, and HTN as present (+) or absent (−), we searched text fields within the participants’ self-reported medical history (RECMHIST.csv and INITHEALTH.csv files downloaded from LONI website at September 12, 2020) using the following criteria to define the presence of these risk factors: smoking: “smok,” diabetes: “diabete,” HLD: “hyperlipidemia” or “'cholesterol,” and HTN: “hypertension” or “HTN” or “high blood pressure.” Cases where “w/o HTN” was noted were designated HTN-.

### CSF Aβ_40_, Aβ_42_, and p-Tau

CSF Aβ_40_, Aβ_42_, and p-Tau_181_ were analyzed by the University of Pennsylvania ADNI Biomarker core laboratory using the fully automated Roche Elecsys and cobas e 601 immunoassay analyzer system [43]. The CSF Aβ_42_/Aβ_40_ and CSF p-Tau/Aβ_40_ ratios were calculated by dividing each CSF measurement by CSF Aβ_40_. We used a Gaussian-mixture model to estimate 2 Gaussian distributions of high CSF Aβ_42_/Aβ_40_ and low CSF Aβ_42_/Aβ_40_ among all 474 (251 CU, 184 MCI, and 39 AD) ADNI participants with CSF Aβ_42_/Aβ_40_ ratio (203 of them were included in this study) and defined an unsupervised threshold of ≤ 0.051 for abnormal CSF Aβ_42_/Aβ_40_, which corresponds to a 90% probability of belonging to the low CSF Aβ_42_/Aβ_40_ distribution (Supplemental fig. 1).

Slopes of CSF Aβ_42_/Aβ_40_ (ΔCSF Aβ_42_/Aβ_40_), CSF Aβ_40_ (ΔCSF Aβ_40_), and CSF p-Tau/Aβ_40_ (ΔCSF p-Tau/Aβ_40_) were calculated based on longitudinal CSF data among 28% of 201 participants (mean of 5.2±2.7 years of follow-up; 2.8±0.7 visits) using a linear mixed-effect (LME) model that included time and a random slope and intercept as independent variables for each participant. We did not adjust for age and sex when we calculated slopes of CSF biomarkers, because we treated them as potential risk factors of CSF biomarkers changes.

### PET imaging and analysis

Details on FBP, FBB, and FDG PET image acquisition and analysis are given elsewhere (http://adni-info.org). Briefly, PET data were acquired in 5-min frames from 50–70 min (FBP), 90–110 min (FBB), and 30–60 min (FDG) post-injection (http://adni-info.org). Pre-processed FBP, FBB, FDG, PET, and structural MRI scans were downloaded from the LONI website (ida.loni.usc.edu). Aβ PET scans were coregistered to the structural MRI scan that was closest in time to the baseline PET. Freesurfer-defined regions of interest (v5.3.0) on structural MRIs were used to extract regional FBP and FBB measurements from the co-registered PET images as described previously [9]. Cross-sectional (at the baseline timepoint) FBP or FBB standardized uptake value ratios (SUVRs) were calculated by dividing uptake across frontal, cingulate, parietal, and temporal regions by that in the whole cerebellum to generate cortical summary SUVRs [44]. Cortical summary SUVR thresholds were defined as FBP ≥1.11 or FBB ≥1.08 as described on the ADNI website (ida.loni.usc.edu). FBP and FBB SUVRs were converted to Centiloids as described previously [9].

FDG PET images for voxel-wise analyses were spatially normalized to the PET template and intensity normalized at the voxel-wise level using the upper 50% of voxels in a pons/vermis reference region [45] using SPM12 (Welcome Department of Imaging Neuroscience, London, UK). These spatially normalized and intensity normalized images were also used to extract FDG SUVRs from a set of predefined and previously validated “metaROIs” (left angular gyrus, right angular gyrus, bilateral posterior cingulate, left inferior temporal gyrus, right inferior temporal gyrus) [46]. FDG SUVR slope (ΔFDG SUVR) was calculated for each participant based on longitudinal FDG data which was available in 19.5% of 201 participants with a mean of 2.9±1.8 years of follow-up (2.2±0.4 visits) using LME model that included time, age, and sex, and a random slope and intercept as independent variables.

### Hippocampal volume and white matter hyperintensities

Hippocampal volume (HCV) (mm^3^) was calculated with Freesurfer and adjusted (aHCV) for intracranial volume (ICV) using the regression approach [47] as described previously [21]. aHCV slope (ΔaHCV) data was available for 30.5% of 201 participants (mean of 5.0±2.6 years of follow-up; 5.8±2.1 visits) using the structural MRI scan that was closest in time to, and after, the baseline CSF p-Tau/Aβ_40_. These slopes were estimated using LME model, including the following independent variables: time, age, and sex, and a random slope and intercept.

WMH measurement was calculated at the University of California, Davis, based on a Bayesian approach to segmentation of high-resolution T1-weighted and FLAIR images as described previously [48] and also on the ADNI website. In order to compensate for individual variance in brain size and non-normal distribution, WMH was normalized to ICV (WMH/ICV) and log_10_ transformed prior to analysis.

### Cognition

Preclinical Alzheimer’s Cognitive Composite (PACC) scores [49] were calculated as described by combing the standard *z* scores (using the mean values of all the ADNI CU participants regardless of amyloid positivity) of the Delayed Recall portion of the Alzheimer’s Disease Assessment Scale, the delayed recall score on the logical memory IIa subtest from the Wechsler Memory Scale, the digit symbol substitution test score from the Wechsler Adult Intelligence Scale–Revised and the MMSE total score. PACC slope (ΔPACC) was calculated based on longitudinal cognitive scores in 37% of 201 participants (mean follow-up=4.8±2.8 years, 5.4±2.4 visits) using LME model, including the following independent variables: time, age, sex, and education and a random slope and intercept.

### Statistical analysis

The normality of distributions was tested using the Shapiro-Wilk test and visual inspection of data. Data are presented as median (interquartile range (IQR)) or number (%) unless otherwise noted. Baseline characteristics were compared between Aβ- CU and CI groups by using a two-tailed Mann-Whitney test or Fisher’s exact test.

**Fig. 1:**
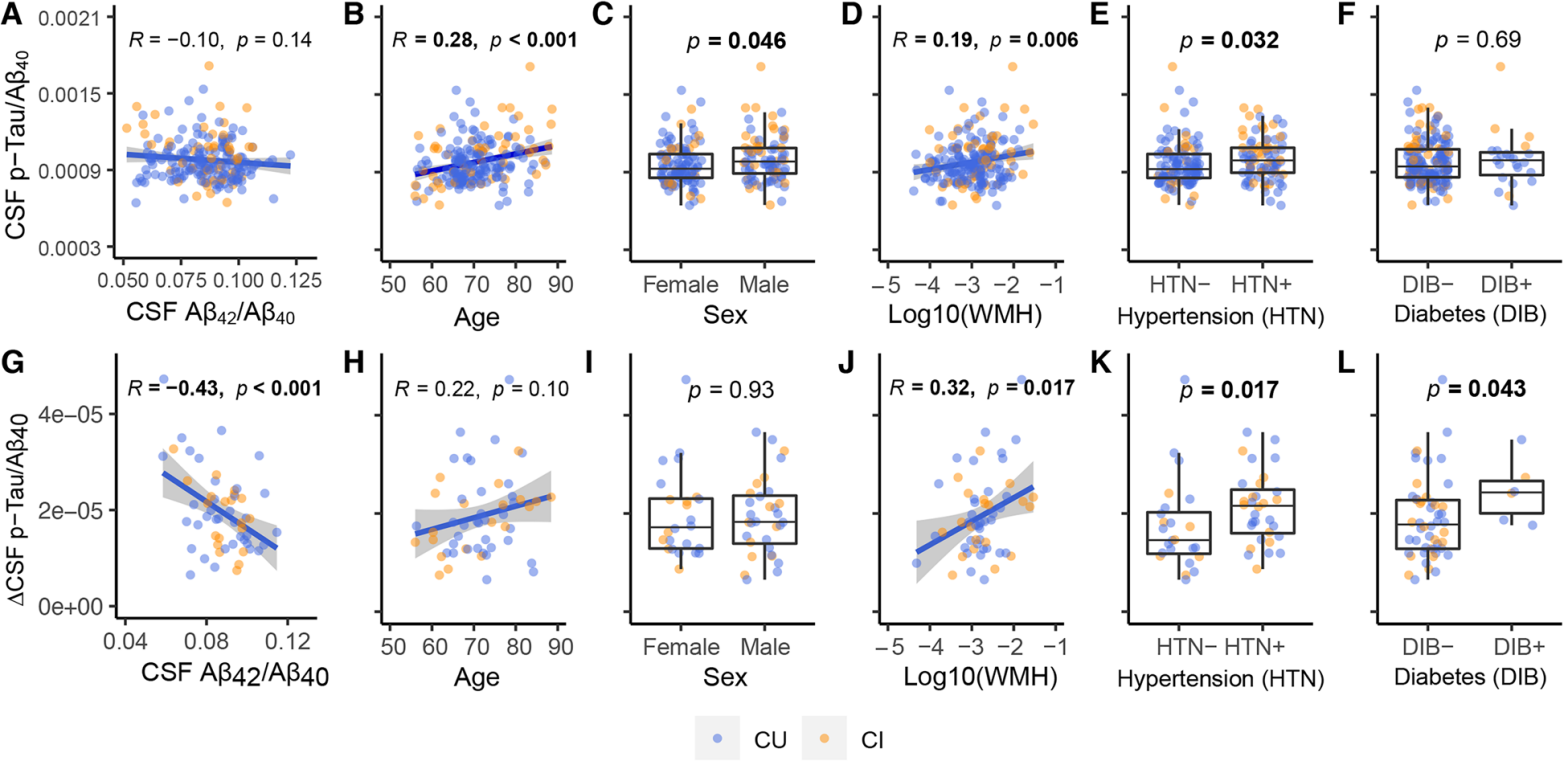
Risk factors related to elevated tau in Aβ- individuals. The associations of risk factors with baseline CSF p-Tau/Aβ_40_ (**A-F**) and longitudinal CSF p-Tau/Aβ_40_ annualized change (**G-L**). Abbreviations: Aβ amyloid-β, CSF cerebrospinal fluid, CU cognitively unimpaired, CI cognitively impaired, p-Tau phosphorylated tau, WMH white matter hyperintensities

In order to determine the factors related to CSF p-Tau/Aβ_40_ increase, we examined the associations of CSF p-Tau/Aβ_40_ and ΔCSF p-Tau/Aβ_40_ with CSF Aβ_42_/Aβ_40_, Aβ PET, age, sex, APOE-ε4 status, WMH, BMI, Smoking, Diabetes, HLD, and HTN using Pearson’s correlation or Mann-Whitney tests. Diabetes was excluded from some following analyses due to limited sample size (Fig. 1). Because CSF p-Tau/Aβ_40_ has been recently validated in Aβ- individuals [21] but is still a relatively novel measurement, we also examined baseline non-ratio CSF p-Tau and ΔCSF p-Tau in the significant associations identified using the ratio CSF p-Tau/Aβ_40_ measurement. We also investigated the prediction of ΔCSF Aβ_42_/Aβ_40_ and ΔCSF Aβ_40_ by baseline CSF p-Tau/Aβ_40_, and its significantly associated risk factors (CSF Aβ_42_/Aβ_40_, WMH, and HTN, see Fig. 1 in the “Results” section). Besides, we used generalized linear models (GLM) models to investigate the associations of CSF p-Tau/Aβ_40_ and ΔCSF p-Tau/Aβ_40_ with their significant risk factors (see Fig. 1 in the “Results” section) together in the same model, respectively.

Associations of voxel-wise FDG PET images with CSF p-Tau/Aβ_40_ and its significant risk factors were analyzed using regression models implemented in SPM12 (Welcome Department of Imaging Neuroscience, London, UK), controlling for age, sex, and diagnosis. Voxel-wise results between CSF p-Tau/Aβ_40_ and FDG PET were presented using an uncorrected voxel threshold of *p* < 0.001. T-maps were converted to R-maps using CAT12 toolbox (www.neuro.uni-jena.de/cat/) and displayed at both without and with family-wise error (FWE) corrected *p* < 0.05 at the cluster level.

We included as covariates in the multivariate analyses only risk factors of CSF p-Tau/Aβ_40_ that had significant associations with FDG SUVR, aHCV, and PACC. We used GLM models to investigate the associations between CSF p-Tau/Aβ_40_, FDG SUVR (metaROIs), and ΔFDG SUVR, controlling for age, sex, and diagnosis. We also investigated the predictive effect of baseline CSF p-Tau/Aβ_40_ on aHCV and ΔaHCV, controlling for HTN, age, sex, and diagnosis. In order to understand the association between aHCV and FDG SUVR, we investigated the predictive effect of baseline FDG SUVR on ΔaHCV controlling for HTN, age, sex, and diagnosis, and baseline aHCV on ΔFDG SUVR controlling for age, sex, and diagnosis.

Finally, we investigated the prediction of ΔPACC with baseline CSF p-Tau/Aβ_40_, FDG SUVR, and aHCV as the predictors, controlling for HTN, age, sex, education, and diagnosis. We also examined the sequential mediation associations between baseline CSF p-Tau/Aβ_40_, aHCV, FDG SUVR, and ΔPACC using latent variable modeling (R; Lavaan package) [50]. CSF p-Tau/Aβ_40_, aHCV, FDG SUVR, and ΔPACC were converted to standard *z* scores. Total, direct, and indirect associations were calculated via a 5000-iteration bootstrapping procedure.

We selected two-sided *p* < 0.05 as the significance level unless otherwise noted. Longitudinal data of biomarkers were defined as the data that was closest in time to, and after, the baseline CSF p-Tau/Aβ_40_. Statistical analyses were performed in the statistical program *R* (v3.6.2, The R Foundation for Statistical Computing) unless otherwise noted.

## Results

### Demographics

Measurements were acquired between July 2010 and August 2020. Demographics of 201 Aβ- participants and the comparisons between CU individuals and CI individuals can be found in Table 1. Longitudinally, 56, 61, 39, and 74 participants had > 2 CSF, aHCV, FDG PET, and PACC cognitive data respectively.

**Table 1:** Demographics of amyloid-negative participants

Diagnosis	Cognitively unimpaired (CU)	Cognitively impaired (CI)
Sample size	149	52
Age (median (IQR))	69.3(7.1)	71.8(16.8)
Education (median (IQR))	18 (2)	16 (5)
Females (no., %)	**95 (63.8%)**^**a**^	20 (38.5%)
APOE4 (no., %)	33 (22.1%)	5 (9.6%)
BMI (median (IQR))	26.7 (6.4)	27.6 (5.0)
Smoking history (no., %)	5 (3.4%)	5 (9.6%)
Diabetes (no., %)	18 (12.1%)	5 (9.6%)
Hyperlipidemia (no., %)	67 (45.0%)	25 (48.1%)
Hypertension (no., %)	54 (36.2%)	**33 (63.5%)**^**b**^
CSF Aβ_42_/Aβ_40_ (median (IQR))	0.086 (0.020)	0.088 (0.014)
Aβ PET centiloid (median (IQR))	4.09 (10.21)	4.02 (14.93)
CSF p-Tau/Aβ_40_ (median (IQR))	0.0009 (0.0002)	**0.0010 (0.0002)** ^**c**^
WMH (median (IQR))	−2.96 (0.67)	−2.87 (0.71) ^d^
PACC (median (IQR))	1.09 (3.61)	−4.65 (4.59) ^e^
**81 participants with aHCV and FDG**		
Sample size	32	49
aHCV (mm^3^) (median (IQR))	7723 (1064)	7319 (2683) ^f^
FDG SUVR (median (IQR))	1.32 (0.09)	1.29 (0.15) ^g^

^a^*p* = 0.002; ^b^*p* = 0.001, Fisher’s exact test; ^c^*p* = 0.030, ^d^*p* = 0.063, ^e^*p* < 0.001, ^f^*p* = 0.054, ^g^*p* = 0.057, Mann-Whitney *U* test

Abbreviations: *Aβ* amyloid-β, *aHCV* adjusted hippocampal volume, *BMI* body mass index, *CSF* cerebrospinal fluid, *FDG* ^18^F-fluorodeoxyglucose, *IQR* interquartile range, p-Tau phosphorylated tau, SUVR standardized uptake value ratio, *WMH* white matter hyperintensities

### Risk factors related to elevated tau in Aβ- individuals

At baseline, elevated CSF p-Tau/Aβ_40_ was unrelated to CSF Aβ_42_/Aβ_40_ but was associated with older age (*R* = 0.28 [95% ci, 0.15, 0.41]), male sex (estimate = 4.2×10^-5^ [95% ci, 6.4×10^-7^, 6.8×10^-5^]), higher WMH (*R* = 0.19 [95% ci, 0.06, 0.32]), and HTN (estimate = 4.1×10^-5^ [95% ci, 2.9×10^-5^, 1.0×10^-4^]) but not diabetes (Fig. 1A–F). When all significant risk factors were entered into a single model, only age was significantly related to CSF p-Tau/Aβ_40_ (Supplemental fig. 2A, standardized *β* (*β*_std_) = 0.22 [95% ci, 0.08, 0.37], *p* = 0.003). None of these significant risk factors (age, sex, WMH, HTN) were related to CSF Aβ_42_/Aβ_40_ or CSF Aβ_40_, and only age was related (*R* = 0.25 [95% ci, 0.11, 0.37], *p* < 0.001) to the non-ratio CSF p-Tau measurement (Supplemental fig. 3). In addition, older age was related to higher WMH (*R* = 0.29 [95% ci, 0.16, 0.42], *p* < 0.001) and HTN (estimate = 3.3 [95% ci, 1.2, 5.3], *p* = 0.001); HTN and WMH were also positively associated (estimate = 0.24 [95% ci, 0.08, 0.39], *p* = 0.005).

CSF Aβ_42_/Aβ_40_ predicted (*R* = −0.43 [95% ci, −0.62, −0.19]) subsequent ΔCSF p-Tau/Aβ_40_ (Fig. 1G), whereas CSF p-Tau/Aβ_40_ was unrelated to ΔCSF Aβ_42_/Aβ_40_ or ΔCSF Aβ_40_ (Supplemental fig. 4). In addition, higher WMH (*R* = 0.32 [95% ci, 0.06, 0.54]) and HTN (estimate = 4.7×10^-6^ [95% ci, 6.2×10^-7^, 8.7×10^-6^]) were associated with subsequent ΔCSF p-Tau/Aβ_40_ (Fig. 1J, K) (but not ΔCSF Aβ_42_/Aβ_40_ or ΔCSF Aβ_40_, Supplemental fig. 4). Individuals with diabetes had faster ΔCSF p-Tau/Aβ_40_ (Fig. 1L), but the small number (*n*=6) of individuals with diabetes limits interpretation. When all the significant risk factors (age, sex, CSF Aβ_42_/Aβ_40_, WMH, and HTN) were entered into a single model, CSF Aβ_42_/Aβ_40_ (*β*_std_ = −0.40 [95%ci, −0.65, −0.14], *p* = 0.003) and HTN (*β*_std_ = 0.51 [95%ci, 0.02, 0.99], *p* = 0.04) were related to ΔCSF p-Tau/Aβ_40_ (Supplemental fig. 2B). Among these significant risk factors related to ΔCSF p-Tau/Aβ_40_, only CSF Aβ_42_/Aβ_40_ was associated (*R* = −0.28 [95% ci, −0.50, −0.02], *p* = 0.038) with the non-ratio ΔCSF p-Tau measurement (Supplemental fig. 4I).

The associations of Aβ PET, APOE-ε4 status, BMI, smoking, diabetes, HLD with either baseline CSF p-Tau/Aβ_40_, or ΔCSF p-Tau/Aβ_40_ were not significant (Supplemental fig. 5).

### Elevated tau and neurodegeneration in Aβ− individuals

**Fig. 2:**
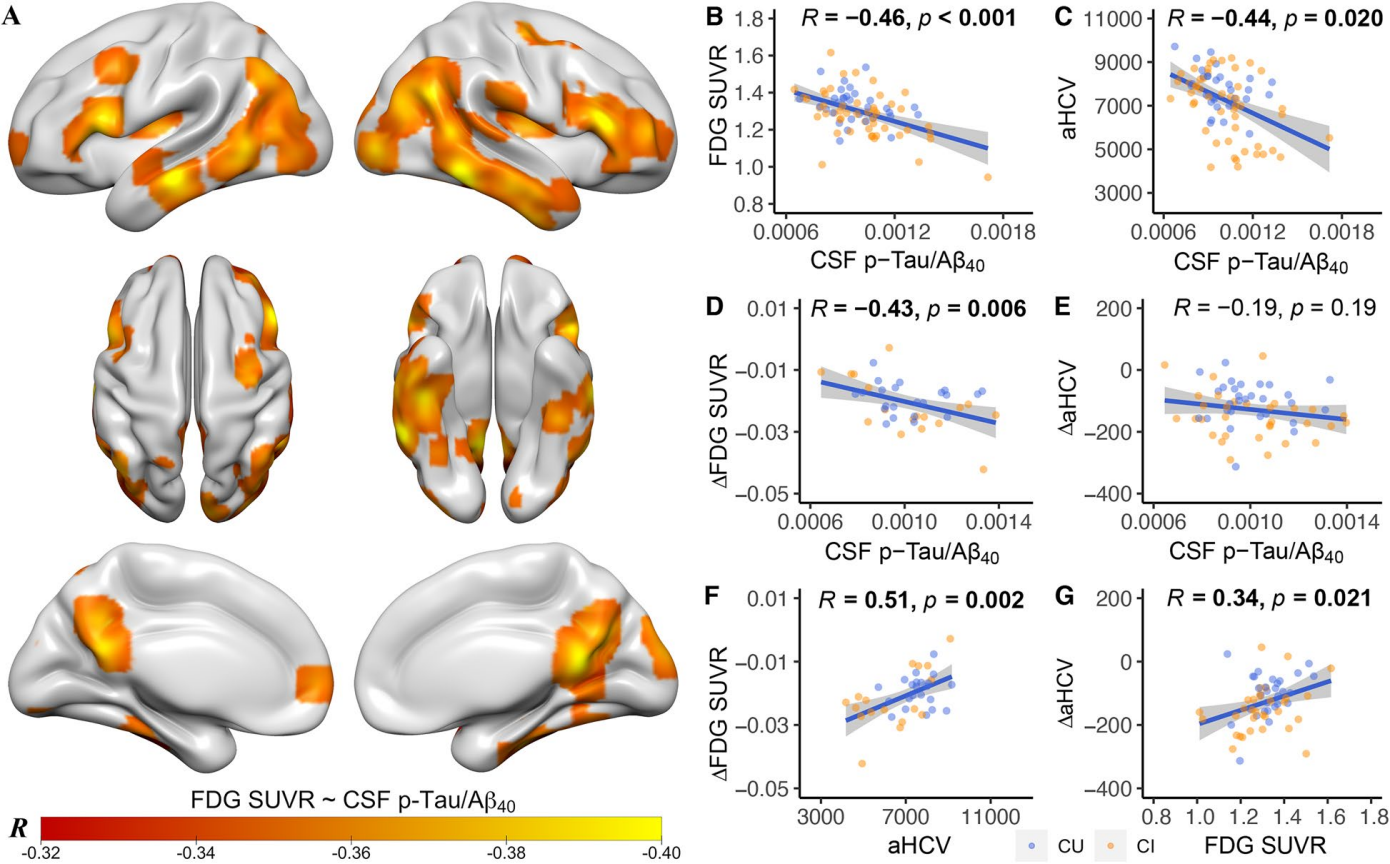
Associations of elevated tau, hypometabolism, and hippocampal atrophy in Aβ- individuals. **A** Voxel-wise correlation between CSF p-Tau/Aβ_40_ and FDG PET. Cross-sectional associations between CSF p-Tau/Aβ_40_, and **B** FDG SUVR (metaROIs) and **C** aHCV. Prediction of ΔFDG SUVR by baseline **D** CSF p-Tau/Aβ_40_ and **F** aHCV. Prediction of ΔaHCV by baseline **E** CSF p-Tau/Aβ_40_ and **G** FDG SUVR (metaROIs). Abbreviations: Aβ amyloid-β, aHCV adjusted hippocampal volume, CSF cerebrospinal fluid, CU cognitively unimpaired, CI cognitively impaired, FDG ^18^F-fluorodeoxyglucose, p-Tau phosphorylated tau, SUVR standardized uptake value ratio

Voxel-wise multiple regression analysis showed that elevated CSF p-Tau/Aβ_40_ was associated with hypometabolism predominantly in inferior temporal, middle temporal, angular gyrus, posterior cingulate/precuneus, inferior parietal, middle frontal, and middle occipital regions (Fig. 2A, *p*<0.001 uncorrected). After FWE correction (*p*<0.05), significant negative associations remained in bilateral posterior cingulate/precuneus, right inferior temporal, middle temporal, and inferior parietal regions (Supplemental fig. 6). Overlap between these results with temporoparietal metaROIs that are characteristic of AD (Supplemental fig. 7) suggests that the metaROI SUVRs are a reasonable measurement of hypometabolism in these Aβ- individuals.

Among these significant risk factors related to CSF p-Tau/Aβ_40_, we found HTN was related to aHCV (*β*_std_ = −0.52 [95% ci, −0.90, −0.14], *p* = 0.009) and ΔPACC (*β*_std_ = −0.46 [95% ci, −0.88, −0.04], *p* = 0.037) (Supplemental fig. 8). Cross-sectionally, CSF p-Tau/Aβ_40_ was related to both FDG SUVR in the metaROIs (Fig. 2B, *β*_std_ = −0.40 [95% ci, −0.62, −0.18]) and aHCV (Fig. 2C, *β*_std_ = −0.25 [95% ci, −0.45, −0.04]). aHCV was correlated with FDG SUVR (*β*_std_ = 0.22 [95% ci, 0.02, 0.42], *R* = 0.38 [95% ci, 0.17, 0.55], *p* = 0.032). Elevated CSF p-Tau/Aβ_40_ predicted subsequent FDG SUVR decrease (Fig. 2D, *β*_std_ = −0.43[95% ci, −0.72, −0.14]), but did not predict change in aHCV (Fig. 2E). Lower aHCV predicted subsequent FDG SUVR decrease (Fig. 2F, *β*_std_ = 0.16 [95% ci, 0.22, 0.85]). Conversely, FDG SUVR also predicted subsequent aHCV decrease (Fig. 2G, *β*_std_ = 0.13 [95% ci, 0.05, 0.54]).

In order to control for the possible influence from longitudinal Aβ changes on our analyses, we also investigated the associations of CSF p-Tau/Aβ_40_, FDG SUVR (metaROIs), aHCV, ΔFDG SUVR, and ΔaHCV after excluding 5 individuals who developed to Aβ+ at follow-up (one MCI changed at CSF Aβ_42_/Aβ_40_ and Aβ PET, 2 CU changed Aβ PET, and 2 CU changed CSF Aβ_42_/Aβ_40_). The results were substantially the same (Supplemental fig. 9).

The baseline non-ratio CSF p-Tau and CSF Aβ_40_ measures were not associated with FDG SUVR (metaROIs), aHCV, ΔFDG SUVR, and ΔaHCV (Supplemental fig. 10).

### Prediction of longitudinal cognitive decline in Aβ- individuals

**Fig. 3:**
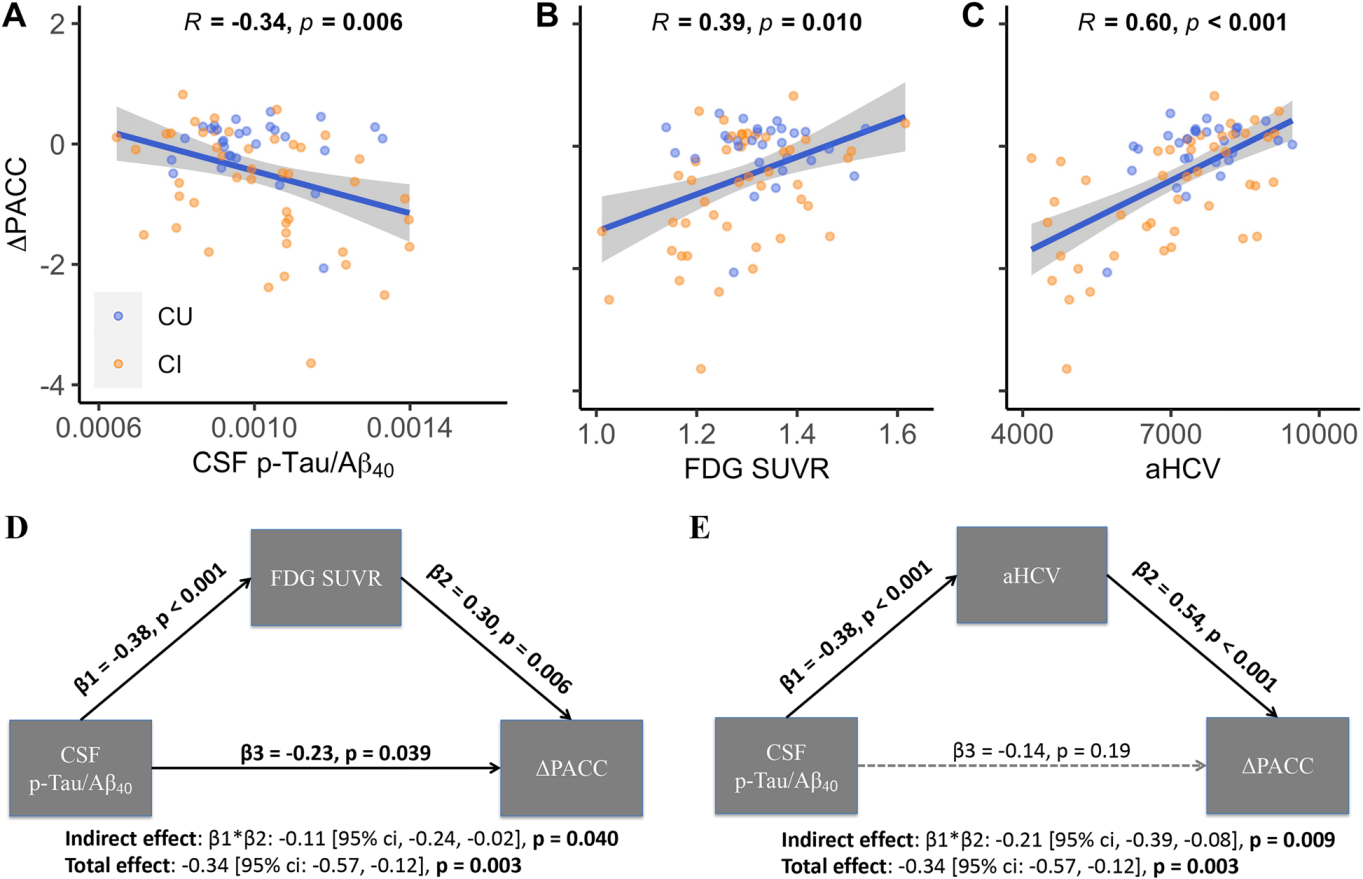
Associations of elevated tau, hypometabolism, hippocampal atrophy, and longitudinal cognitive decline in Aβ- individuals. Prediction of ΔPACC by baseline **A** CSF p-Tau/Aβ_40_, **B** FDG SUVR, and **C** aHCV. **D** Pathways between CSF p-Tau/Aβ_40_, FDG SUVR, and ΔPACC. **E** Pathways between CSF p-Tau/Aβ_40_, aHCV, and ΔPACC. Abbreviations: Aβ amyloid-β, aHCV adjusted hippocampal volume, CSF cerebrospinal fluid, CU cognitively unimpaired, CI cognitively impaired, FDG ^18^F-fluorodeoxyglucose, PACC Preclinical Alzheimer Cognitive Composite, p-Tau phosphorylated tau, SUVR standardized uptake value ratio

The median (IQR) annual ΔPACC of CI individuals (−0.56 (1.47)) was faster (estimate = −0.54 [95% ci, −0.97, 0.18], *p*<0.001) than CU individuals (0.09 (0.46)). CSF p-Tau/Aβ_40_ (Fig. 3A, *β*_std_ = −0.29 [95% ci, −0.50, −0.09]), FDG SUVR (metaROIs) (Fig. 3B, *β*_std_ = 0.28 [95% ci, 0.07, 0.50]), and aHCV (Fig. 3C, *β*_std_ = 0.52 [95% ci, 0.33, 0.72]) all predicted subsequent cognitive decline (ΔPACC). The baseline non-ratio CSF p-Tau and CSF Aβ_40_ measures were not associated with ΔPACC (Supplemental fig. 10).

**Fig. 4:**
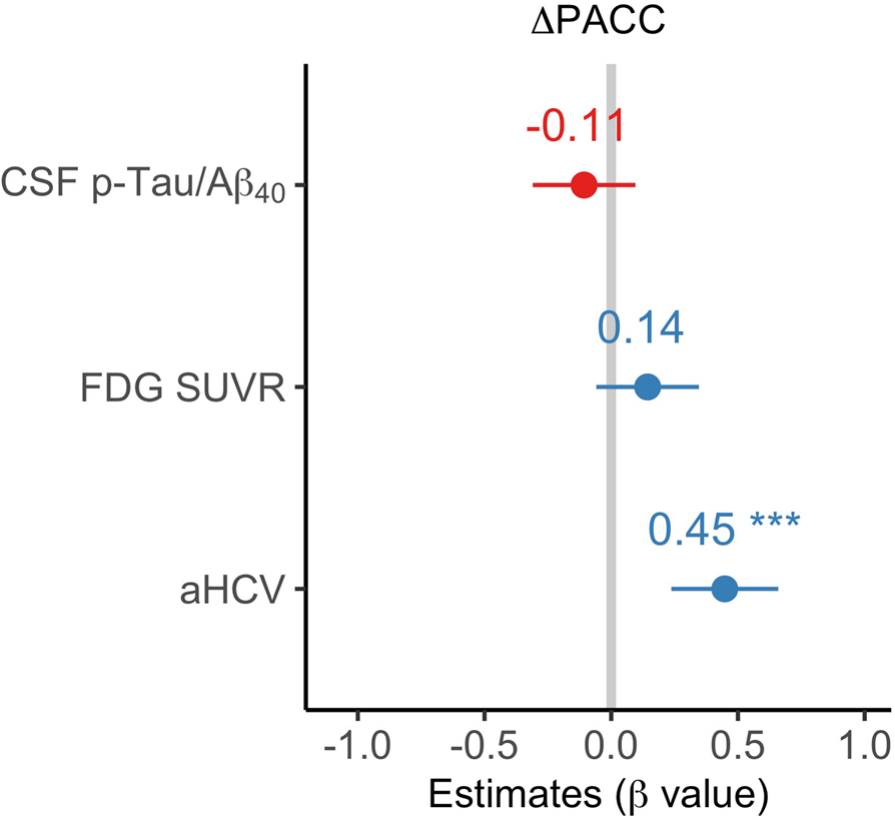
Prediction of longitudinal cognitive decline (ΔPACC) by baseline CSF p-Tau/Aβ_40_, FDG SUVR, and aHCV in one multivariate model. Abbreviations: Aβ amyloid-β, aHCV adjusted hippocampal volume, CSF cerebrospinal fluid, CU cognitively unimpaired, CI cognitively impaired, FDG ^18^F-fluorodeoxyglucose, PACC Preclinical Alzheimer Cognitive Composite, p-Tau phosphorylated tau, SUVR standardized uptake value ratio

In the mediation analysis, FDG SUVR (Fig. 3D) partially and aHCV (Fig. 3E) fully mediated the association between CSF p-Tau/Aβ_40_ and ΔPACC. Furthermore, only aHCV was significantly (*β* = 0.0003[95% ci: 0.0002, 0.0004]) related to longitudinal PACC decline when all the predictors were entered into one model (Fig. 4).

## Discussion

In this study, we investigated a broad range of biomarker, cerebrovascular, and cognitive associations with tau in elderly adults who were unambiguously Aβ- on the basis of both CSF and PET measurements at baseline and therefore seemingly unlikely to be on the pathway to AD. In these individuals, elevated tau measured with CSF was associated with several risk factors (older age, male sex, greater WMH burden, and hypertension), as well as a pattern of neurodegeneration that is characteristic of AD (temporoparietal hypometabolism, and hippocampal atrophy). In longitudinal analyses, lower CSF Aβ_42_/Aβ_40_, higher WMH, and hypertension at baseline predicted tau increases over about 5 years of follow-up. Elevated baseline tau was also associated with subsequent cognitive decline, which was partially mediated by temporoparietal hypometabolism but fully mediated by hippocampal atrophy. These relationships between tau, cerebrovascular disease, and neurodegeneration biomarkers that are typical of AD raise interesting questions about non-amyloid pathways that may be important in the etiology of cognitive decline.

Although there was no relationship between CSF Aβ_42_/Aβ_40_ and CSF p-Tau/Aβ_40_ at baseline, lower CSF Aβ_42_/Aβ_40_ predicted subsequent longitudinal CSF p-Tau/Aβ_40_ increase but not vice versa. There was thus no countervailing evidence that tau deposition drives changes in Aβ in these data or in a previous report from our laboratory [9]. These findings seem most compatible with the hypothesis that, at least in part, Aβ pathology can drive changes in tau even when Aβ is in a “normal” range. Other data have shown significant CSF p-Tau increases in Aβ PET negative individuals [20, 22], and our data extend these results by indicating that Aβ and tau are positively associated with one another prior to reaching the positivity thresholds of both CSF Aβ and Aβ PET. Together with other studies reporting significant Aβ-related tau deposition and cognitive decline in Aβ PET negative cognitively healthy individuals [51–55], these data indicate that tau can begin depositing in nominally “Aβ- individuals.”

One of the key findings in this study was that white matter lesions and hypertension were related to both baseline and longitudinal CSF p-Tau/Aβ_40_ increases in the absence of abnormal Aβ pathology whereas they had no association with CSF Aβ_42_/Aβ_40_ or Aβ_40_ alone, suggesting vascular risk factors may be closely linked to CSF p-Tau/Aβ_40_ independent of Aβ pathology. ADNI data have previously been explored to examine some of the relationships between tau and the variables we measured, though usually not in Aβ- individuals. For example, a few studies based on the ADNI cohort reported significant associations of CSF p-Tau or plasma p-Tau with WMH [25, 37, 56] and hypertension [41]. In addition, some non-ADNI studies reported similar findings although without specifically examining relationships in Aβ- individuals. For instance, a few studies found CSF p-Tau was associated with WMH [38], global mean diffusivity in white matter [57], and hypertension [40]; two PET studies found PET measures of brain tau [58, 59] were related to vascular health variables. In addition, neuropathological studies also found that higher WMH were related to greater neurofibrillary tangles [60] or both tangles and plaques [61], and higher blood pressure was related to higher number of neurofibrillary tangles [62] or both plaques and tangles [63].

Age is a well-known risk factor for tau accumulation, and it was also related to these vascular factors in our data. In a multivariable model, age was the only variable associated with tau. However, both WMH and HTN, but not age, were related to longitudinal tau increases. Together, these findings suggest that both age and cerebrovascular risk factors may be related to tau increases in the absence of abnormal Aβ pathology.

A surprising result of this study was the association between tau and regional cerebral hypometabolism in a pattern typically associated with AD despite the absence of measurable Aβ. In patients with clinical and biomarker evidence of AD, patterns of tau deposition are highly correlated with glucose metabolism while Aβ deposition is not [64]. Although we do not know the level of cortical tau deposition in our subjects because CSF measurements of tau cannot provide regional information, these results are suggestive of tau deposition affecting regional metabolism in a topography that recapitulates AD, suggesting that tau-related, AD-like neurodegeneration can occur in a setting of low Aβ.

Further evidence that elevated tau is related to neurodegeneration is found in its relationship with hippocampal volume. In fact, all three variables–tau, glucose metabolism in typically affected AD regions, and hippocampal volume–were related to one another at baseline and to a large extent in longitudinal relationships (although baseline p-Tau/Aβ_40_ predicted metabolic decline but not progressive atrophy). Previous work is inconsistent, with some studies of Aβ- ADNI participants failing to find associations between CSF p-Tau and hippocampal or cortical atrophy [24, 25], while others find significant relationships between CSF p-Tau and reduced cortical thickness independent of Aβ pathology [23]. It is difficult to understand how opposite findings can arise from the same study, although differences in the sample (such as the sample size and/or the proportion with cognitive impairment), CSF assays, and our use of the normalizing Aβ_40_ ratio [21] may play a role. Regardless of these discrepancies, our data seem to provide strong evidence for an Alzheimer’s-type neurodegeneration that is progressive and related to tau even in amyloid negative individuals.

In addition to its relationship with neurodegeneration, tau was also related to cognitive decline, providing further evidence that it is not benign when Aβ is low. These “downstream” pathways appear to be related as tau affects cognition through both glucose metabolism and hippocampal atrophy. Previous studies have not been in agreement that elevated CSF p-Tau in Aβ- individuals is linked to significant cognitive decline [26, 65], although our sample was older and included those with cognitive impairment.

### Limitations

This study has several limitations. Our findings were based on tau measured using CSF p-Tau_181_/Aβ_40_, but it would be helpful to validate the findings in other samples and with other phosphorylation sites [66]. Furthermore, it is possible that the use of the p-Tau_181_/Aβ_40_ ratio introduces a confounder that could account for these associations, we think that this is unlikely since CSF Aβ_40_ alone was not associated with any of the factors we investigated. Therefore, we believe that these findings reinforce earlier evidence [21] that there is considerable noise among Aβ-negative individuals in CSF p-Tau_181_ measurements due to individual variability in CSF production and due to the limited dynamic range in this group, and this variability can be attenuated by adjusting for Aβ_40_. This noise reduction appears to facilitate the observation of subtle associations between tau and other risk factors at early stages of abnormality. An additional limitation of this study was that some Aβ- subgroups we examined were very small, such as APOE-ε4 carriers, which limited our ability to examine covariates and other risk factors. Finally, tau PET is a relatively late addition to ADNI, so we did not include this because of minimal longitudinal tau data. Nevertheless, CSF p-Tau may have been an advantageous measure for the scientific questions in this study because it probably reflects earlier tau pathology than PET [20, 21].

## Conclusions

The elevation of p-Tau in CSF in our Aβ- subjects is consistent, at least nosologically, with the disorder that has been described as PART; however, in the absence of neuropathology we cannot confirm this diagnosis. These data provide surprising clues as to the etiology and significance of tau pathology in aging. It seems likely that, in addition to age, both cerebrovascular disease and subthreshold levels of Aβ are related to this tau accumulation. Whether cerebrovascular factors alone or in concert with low levels of Aβ can drive tau pathology is uncertain, but the literature is replete with evidence for a relationship between tau and cerebrovascular disease. Crucially, evidence of this tau pathology occurs early, as CSF probably reflects tau abnormalities before PET scans do, and shares features with AD such as a pattern of metabolic decline and regional brain atrophy. Further longitudinal follow-up of these individuals will be critical for determining whether these processes are Alzheimer’s-independent. However, our data indicate that this phenotype of Aβ- CSF tau elevation is similar to AD, which implies that it may represent an amyloid-independent pathway to AD, a pathophysiology that mimics AD, or an important, and very early, interaction between Aβ and vascular disease that underlies neurodegeneration and cognitive decline.

## Supplementary Information

**Additional file 1.** Supplemental material can be found at online.

## Data Availability

The dataset supporting the conclusions of this article is available in the ADNI repository (ida.loni.usc.edu). Derived data is available from the corresponding author on request by any qualified investigator subject to a data use agreement.

## Abbreviations

Aβ: Amyloid-β
AD: Alzheimer’s disease
ADNI: Alzheimer’s Disease Neuroimaging Initiative
aHCV: Adjusted hippocampal volume
BMI: Body mass index
β_std_: Standardized β coefficient
CI: Confidence interval
CSF: Cerebrospinal fluid
CU: Cognitively unimpaired
FBB: ^18^F-florbetaben
FBP: ^18^F-florbetapir
FDG: ^18^F-fluorodeoxyglucose
GLM: Generalized linear model
HLD: Hyperlipidemia
HCV: Hippocampal volume
HTN: Hypertension
ICV: Intracranial volume
LME: Linear mixed-effect
MCI: Mild cognitive impairment
PACC: Preclinical Alzheimer Cognitive Composite
PART: Primary-Age Related Tauopathy
p-Tau: phosphorylated tau
ROI: Regions of interest
SUVR: Standardized uptake value ratio
WMH: White matter hyperintensities
