# Supplementary material for: Age, vascular disease, and Alzheimer’s disease pathologies in amyloid negative elderly adults
Source: Alzheimers Res Ther. 2021 Oct 15;13:174. doi: 10.1186/s13195-021-00913-5 (PMC8520216; doi:10.1186/s13195-021-00913-5)
Supplement: Supplementary file 1 — Additional file 1. Supplemental material can be found at online. [file 13195_2021_913_MOESM1_ESM.pdf]

Supplemental Material

Cutoff of CSF Aβ$_{42}$/Aβ$_{40}$

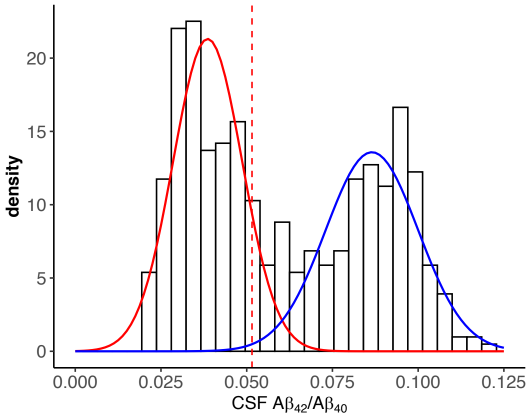

Supplemental fig.1. *Estimates of 2 gaussian distributions of high CSF Aβ$_{42}$/Aβ$_{40}$ (blue curve) and low CSF Aβ$_{42}$/Aβ$_{40}$ (red curve) of among all 474 (251 CU, 184 MCI and 39 AD) ADNI participants with CSF Aβ$_{42}$/Aβ$_{40}$ ratio. Red dashed vertical line reflects the Aβ+ threshold of CSF Aβ$_{42}$/Aβ$_{40}$ ratio 0.051, which corresponds to a 90% probability of belonging to the low CSF Aβ$_{42}$/Aβ$_{40}$ distribution.*

Supplemental table1. Demographics of longitudinal data

Diagnosis	Cognitively unimpaired	Cognitively impaired
**56 participants with ≥ 2 CSF measurements**		
Sample size	36	20
Duration of follow-up, year, (Mean (SD))	5.9 (2.9)	3.9 (1.8)
Visits of CSF, No., (Mean (SD))	2.8 (0.6)	2.8 (0.9)
**61 participants with ≥ 2 aHCV**		
Sample size	29	32
Duration of follow-up, year, (Mean (SD))	6.5(1.7)	3.8(2.6)
Visits of aHCV, (Mean (SD))	6.4(1.5)	5.2(2.5)
**39 participants with ≥ 2 FDG PET**		
Sample size	22	17
Duration of follow-up, year, (Mean (SD))	2.4(1.4)	3.7(2.1)
Visits of FDG PET, (Mean (SD))	2.1(0.3)	2.3(0.5)
**74 participants with ≥ 2 cognition**		
Sample size	29	45
Duration of follow-up, year, (Mean (SD))	6.8(1.3)	3.5(2.7)
Visits of PACC scores, (Mean (SD))	6.4(1.0)	4.6(2.8)

The associations of age, vascular disease and CSF Aβ$_{40}$, CSF Aβ$_{42}$/Aβ$_{40}$ and CSF p-Tau/Aβ$_{40}$

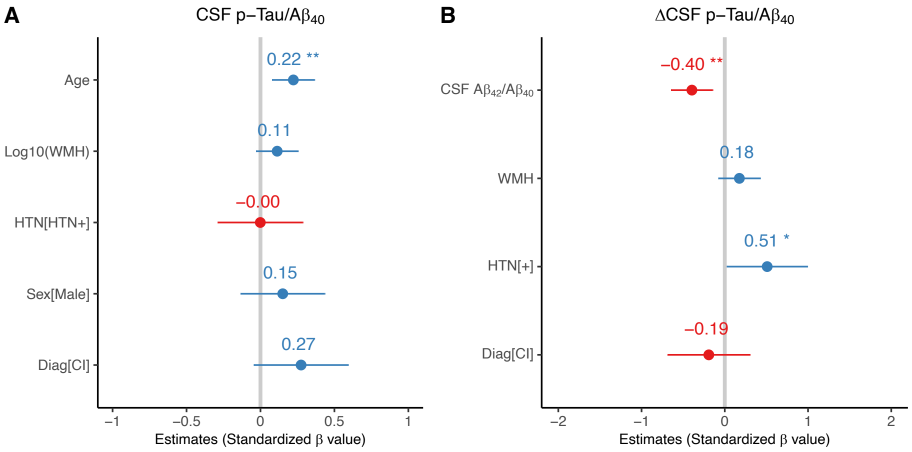

Supplemental fig. 2*. (A). Associations of baseline CSF p-Tau/Aβ$_{40}$ with age, white matter hyperintensities (WMH), hypertension (HTN), sex and diagnosis in one model. (B). Associations of longitudinal CSF p-Tau/Aβ$_{40}$ changes (ΔCSF p-Tau/Aβ$_{40}$) with CSF Aβ$_{42}$/Aβ$_{40}$, WMH, HTN and diagnosis in one model.*

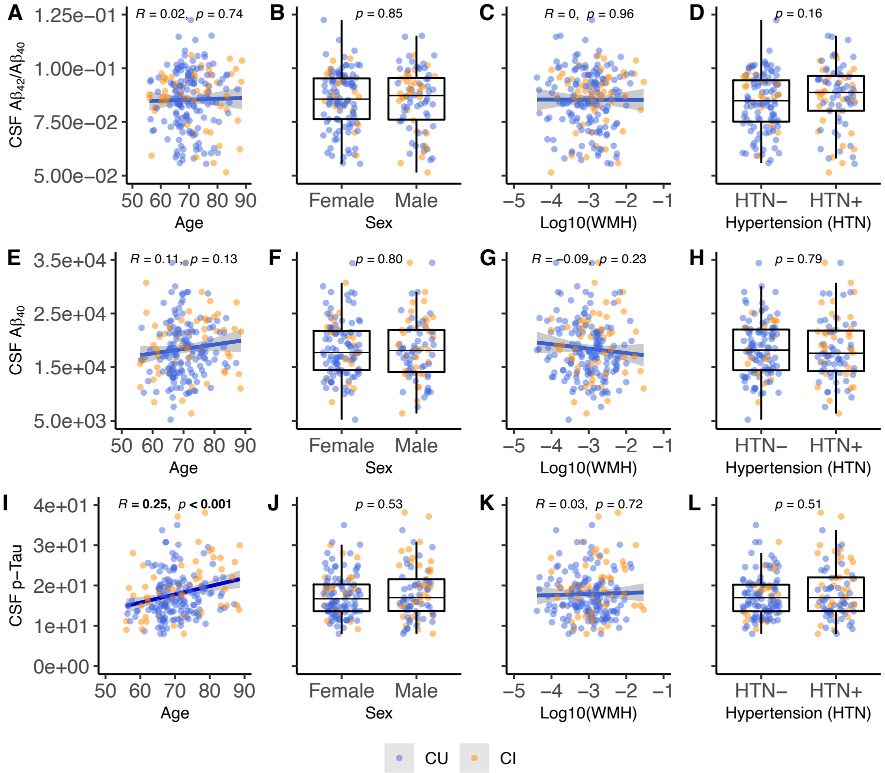

Supplemental fig. 3. *The association of age, sex, WMH and HTN with (A-D) CSF Aβ$_{42}$/Aβ$_{40}$, (E-H) CSF Aβ$_{40}$ and (I-L) CSF p-Tau alone in Aβ- individuals.*

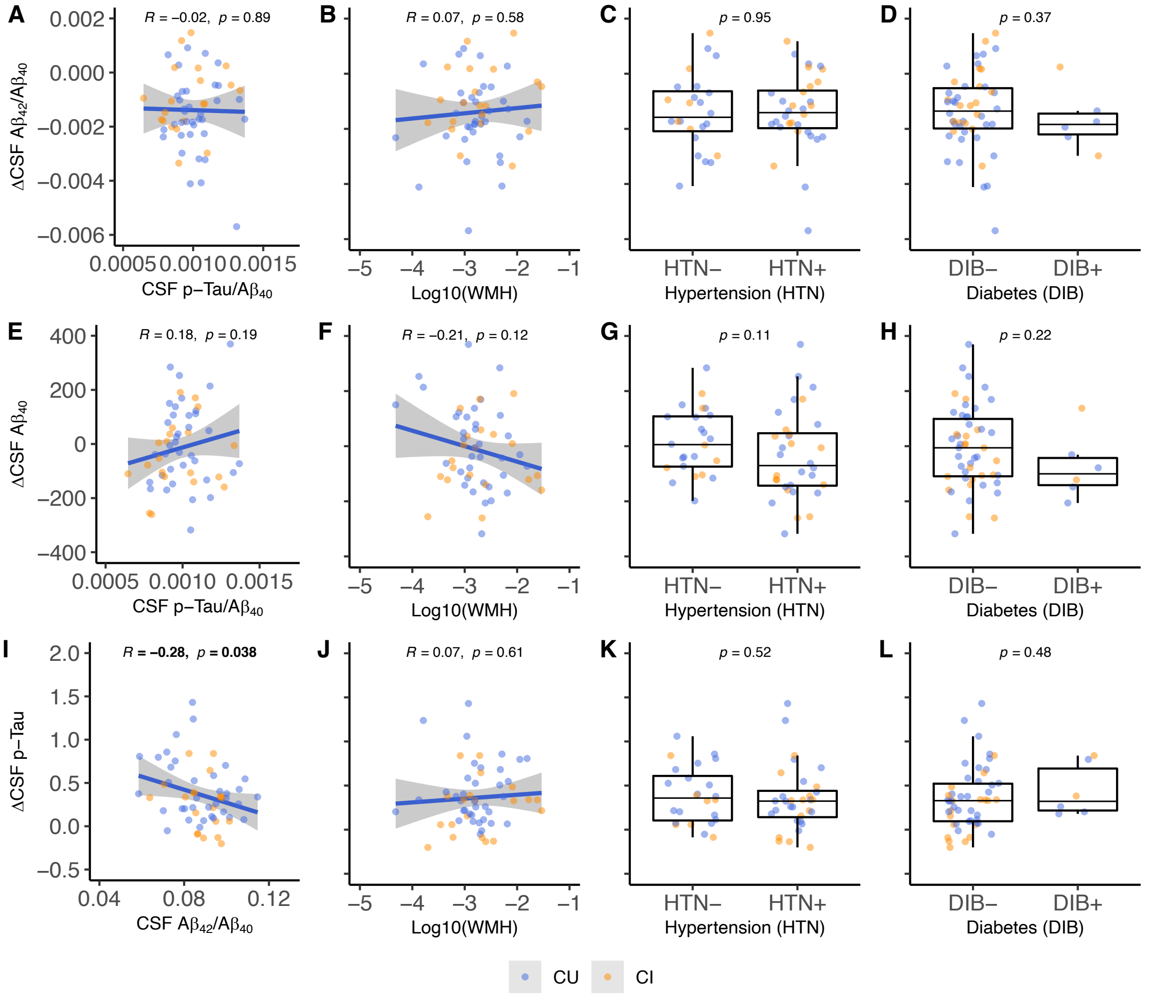

Supplemental fig. 4. *The association of age, sex, WMH and HTN with (A-D) ΔCSF $A\beta_{42}/A\beta_{40}$, (E-H) ΔCSF $A\beta_{40}$ and (I-L) ΔCSF p-Tau alone in Aβ- individuals.*

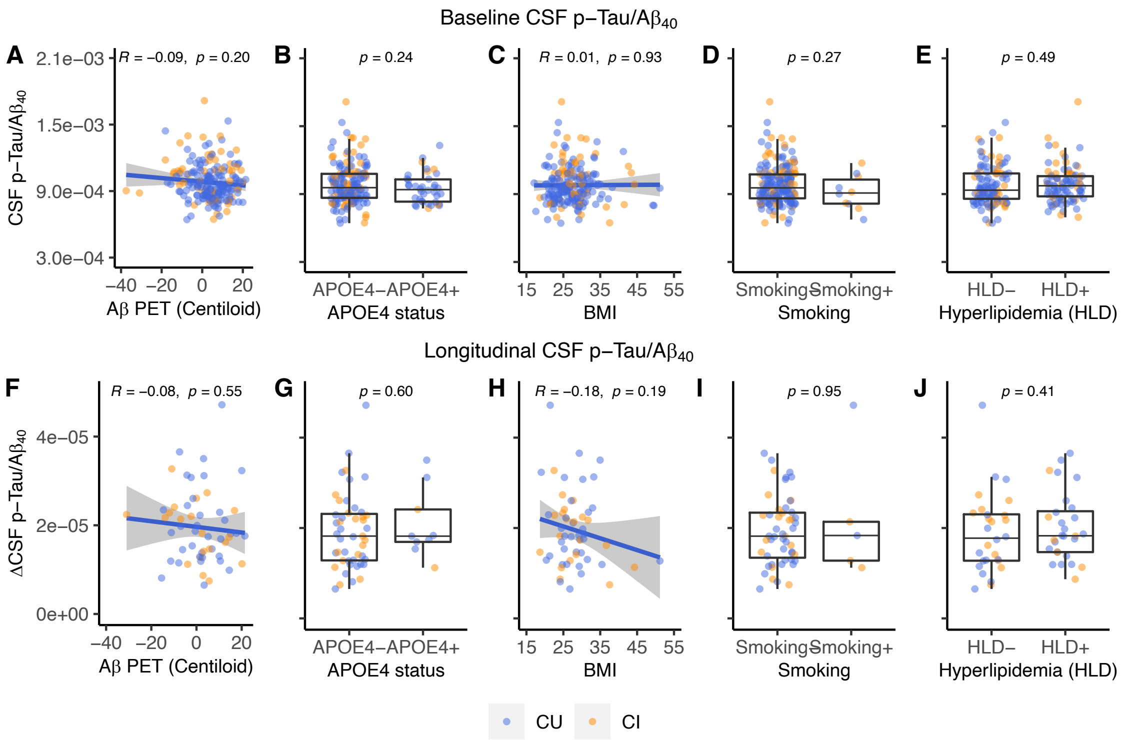

Supplemental fig. 5. Risk factors not related to CSF p-Tau/Aβ$_{40}$ increase in Aβ- individuals. *The associations of risk factors with (A-E) baseline CSF p-Tau/Aβ$_{40}$ and (F-J) longitudinal CSF p-Tau/Aβ$_{40}$ changes (ΔCSF p-Tau/Aβ$_{40}$).*

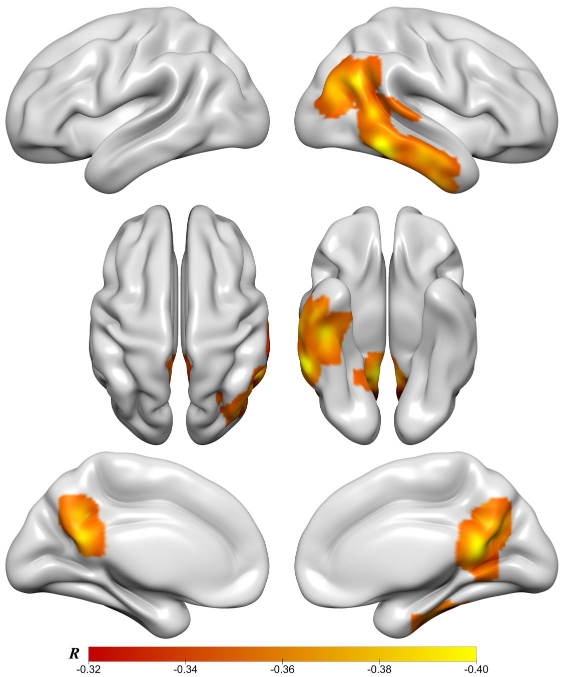

Supplemental fig. 6. *Voxel-wise correlation between CSF p-Tau/Aβ$_{40}$ and FDG PET with family-wise error (FWE) corrected p < 0.05 at cluster level.*

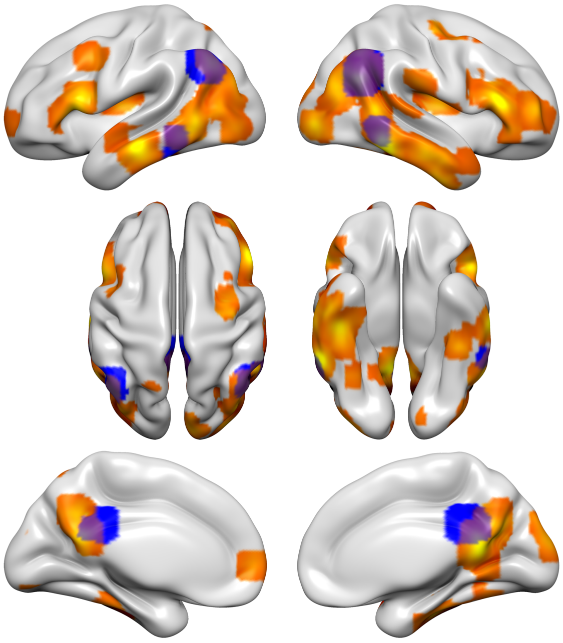

Supplemental fig. 7. *Overlap (purple) of significant brain regions (red-yellow) between FDG SUVR and CSF p-Tau/Aβ$_{40}$ with Alzheimer's metaROI (left angular gyrus, right angular gyrus, bilateral posterior cingulate, left inferior temporal gyrus, right inferior temporal gyrus) region (blue)of hypometabolism.*

Associations of CSF Aβ_{42}/Aβ_{40}, WMH, HTN, aHCV and ΔPACC

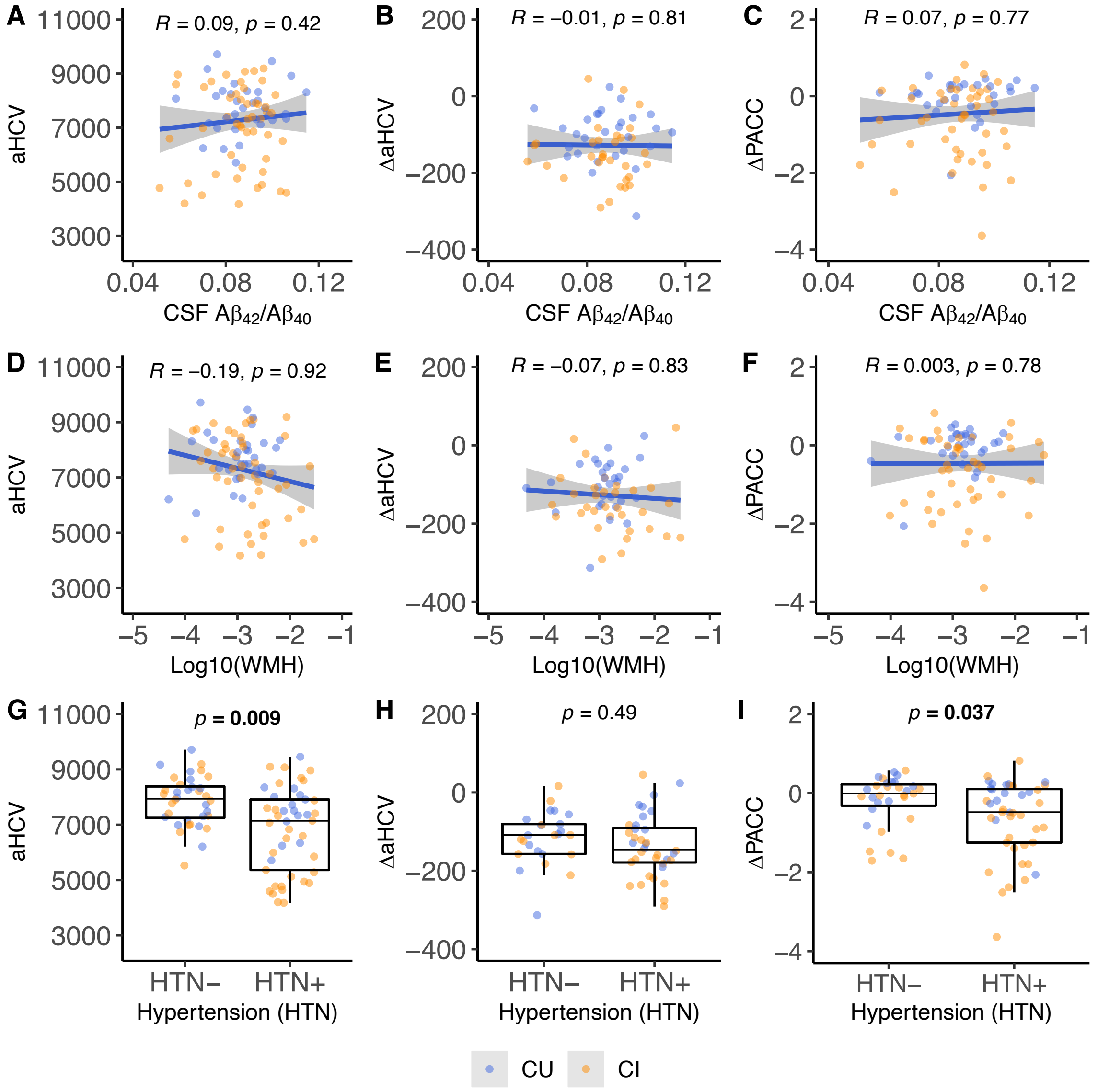

Supplemental fig. 8. *Associations of Aβ_{42}/Aβ_{40}, White Matter Hypertension (WMH), Hypertension (HTN), adjusted hippocampal volume (aHCV) and PACC Slope (ΔPACC).*

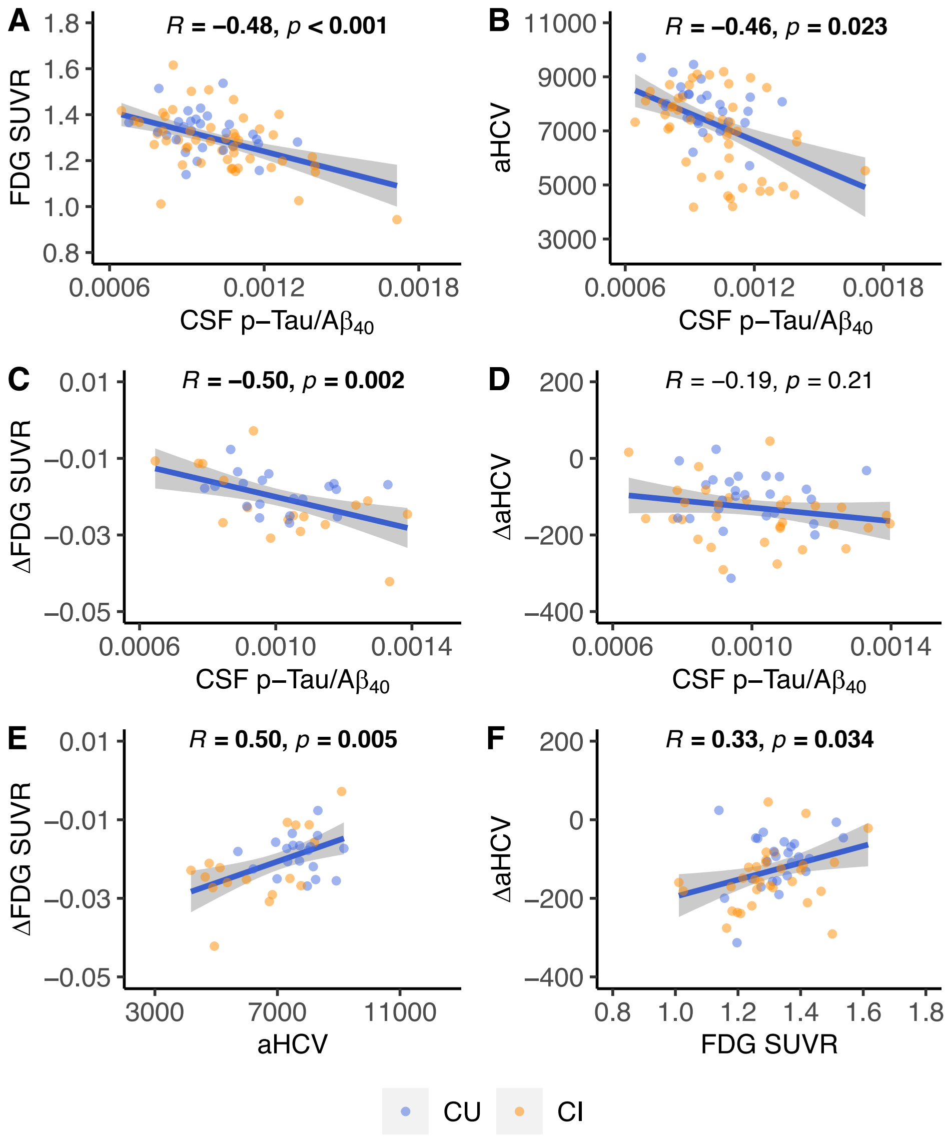

Supplemental fig. 9. Associations of elevated tau, hypometabolism and hippocampal atrophy in Aβ- individuals after excluding Aβ status converters*. Cross-sectional associations between CSF p-Tau/Aβ$_{40}$, and (A) FDG SUVR (metaROIs) and (B)aHCV. Prediction of ΔFDG SUVR by baseline (C) CSF p-Tau/Aβ$_{40}$, and (E) aHCV. Prediction of ΔaHCV by baseline (D) CSF p-Tau/Aβ$_{40}$, and (F) FDG SUVR (metaROIs).*

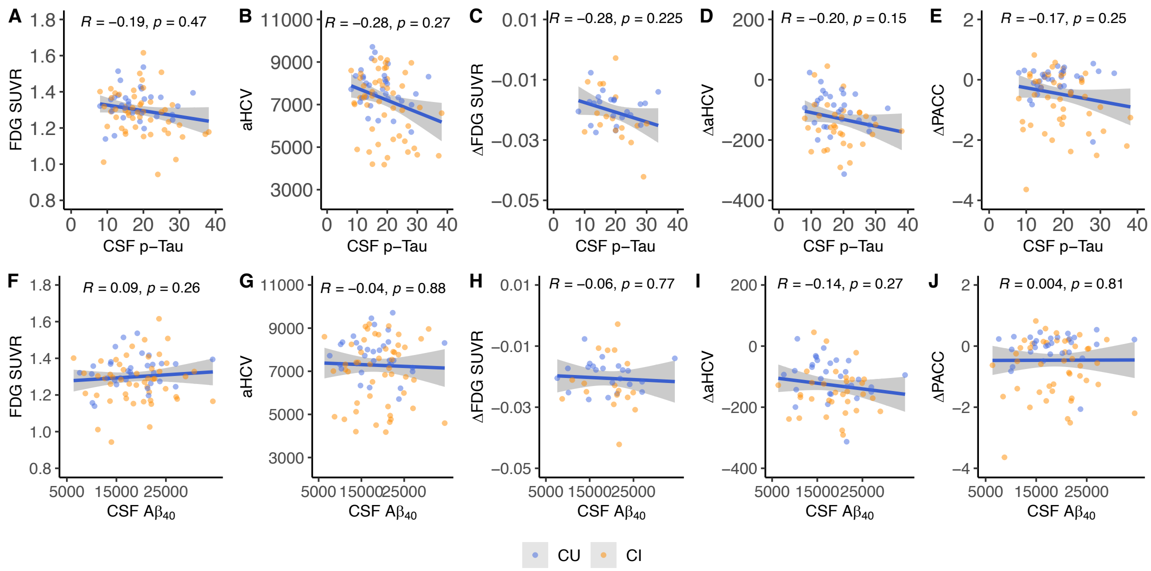

***Supplemental fig. 10. Associations of CSF p-Tau alone, CSF Aβ$_{40}$, hypometabolism, hippocampal atrophy and cognitive decline in Aβ- individuals**. Cross-sectional associations between CSF p-Tau, and (A) FDG SUVR (metaROIs) and (B)aHCV. Prediction of (C) ΔFDG SUVR (metaROIs), (D) ΔaHCV and (E) ΔPACC by baseline CSF p-Tau. Cross-sectional associations between CSF Aβ$_{40}$, and (F) FDG SUVR (metaROIs) and (G)aHCV. Prediction of (H) ΔFDG SUVR (metaROIs), (I) ΔaHCV and (J) ΔPACC by baseline CSF Aβ$_{40}$.*
